# Cone Beam Computed Tomography Study of Root Canal Morphology of Permanent Mandibular Incisors in Indian Subpopulation

**DOI:** 10.12659/PJR.901840

**Published:** 2017-07-07

**Authors:** Gaurav Ravishankar Verma, Chetan Bhadage, Ajay R. Bhoosreddy, Priyanka R. Vedpathak, Gayatri P. Mehrotra, Ashwini C. Nerkar, Akanksha Bhandari, Shweta Chaubey

**Affiliations:** 1 Department of Oral Medicine and Radiology, Mahatma Gandhi Vidyamandir’s Karmaveer Bhausaheb Hire Dental College and Hospital, Nashik, Maharashtra, India; 2 Department of Paediatric and Preventive Dentistry, Oxford Dental College, Bangalore, Karnataka, India

**Keywords:** Cone-Beam Computed Tomography, Endodontics, Tooth Abnormalities

## Abstract

**Background:**

The aim of the study was to determine the root canal morphology of permanent mandibular incisor teeth in the Indian subpopulation with the use of cone beam computed tomography (CBCT).

**Material/Methods:**

CBCT images of 200 patients with 800 permanent mandibular incisors, fulfilling necessary inclusion criteria and aged 18 to 60 years were evaluated. The number of roots, number of root canals and canal configuration were investigated and then classified according to Vertucci’s classification of root canals. The effect of gender on the incidence of root canal morphology was also investigated.

**Results:**

All the permanent mandibular incisors had a single root. The majority of mandibular incisors (66.5%) had a single root with a single canal. The prevalence of second canals was as follows: right central incisor – 33.5%, left central incisor – 30%, right lateral incisors – 33.5% and left lateral incisor – 36.5%. According to gender, 15.2% of men and 20.4% of women had a second root canal. Type 1 Vertucci configuration was most prevalent, followed by type 3, type 2, type 5 and type 4 in that order.

**Conclusions:**

Type 1 Vertucci’s classification (64.5%) was the most prevalent canal configuration in the mandibular anterior teeth in the Indian population. Type 5 Vertucci’s classification was the most frequently observed canal configuration of the two-canalled teeth. CBCT is an excellent imaging modality for detection of different canal configurations of mandibular incisors.

## Background

Appropriate knowledge of root canal morphology is very important, because an accurate diagnosis leads to favorable endodontic treatment outcomes. Root canal morphology varies among different ethnic populations, because it is thought to be racially and genetically determined. One of the main reasons for root canal treatment failure is the lack of knowledge of root canal morphology.

In permanent mandibular incisors, the most common form is a single root with a single root canal [1]. It was believed earlier that mandibular anterior teeth have only one root canal [2]. However, root canal morphology of permanent mandibular incisor is not simple, as it may be complicated by the presence of second canal, lateral canal and apical deltas [3]. Lack of identification of anatomical variations or additional canal may lead to endodontic treatment failure.

Different techniques have been previously used to study the root canal morphology of permanent mandibular incisors such as canal staining and clearing technique [1], conventional radiography [4], contrast-enhanced radiography [5], modified canal staining and clearing [6] and computed tomography. Conventional periapical radiograph is a very important tool for identification of root canal morphology *in vivo* [7]. However, such radiographs are not completely reliable, because there are inherent shortcomings such as overlapping of bony and dental structures and distortion [8].

Recently, cone beam computed tomography (CBCT) has been introduced into dental imaging, which provides three dimensional evaluations of dental and maxillofacial structures. CBCT explains the internal structure of an object with the use of cone-shaped beams of radiation that can acquire data in a single 360-degree rotation.

When CBCT is compared with conventional periapical radiograph, it has a combination of axial, coronal and sagittal sections, which minimizes distortion and overlapping of anatomical structures. When CBCT is compared with conventional computed tomography (CT), it provides less radiation, lower scan time and it also increases accuracy and resolution [9].

Review of the literature reveals that CBCT for the assessment of root canal morphology of permanent mandibular incisors is mainly used in Europe, North America, Japan, Taiwan, China and Iran [10–12], while the studies in the Indian population are limited. The aims of present study were as follows:

1. To investigate the configuration and morphology of the root canal system of mandibular central and lateral incisors with the use of CBCT.
2. To investigate the incidence of second canal in permanent mandibular incisors in men and women

## Material and Methods

The study was conducted in the Department of Oral Medicine and Radiology, MGV’S Dental College and Hospital, Nashik, India. The CBCT images were obtained using CBCT dental imaging system (Galileos, Sirona system, Bensheim, Germany) operating at 98 kvp and 5–15 mA. The gender of patients was also recorded.

CBCT images of 800 permanent mandibular incisors were included that fulfilled the following inclusion criteria:

1. No obvious dental caries.
2. Fully developed root canal apices without resorptions and calcification.
3. CBCT images of good quality with desired area of interest.
4. Absence of canal filling, post and crown restoration.

The root and root canal morphology of permanent mandibular incisors were assessed in axial, sagittal and horizontal sections by two investigators. Two investigators examined the image separately and any difference between them was discussed until a mutual agreement was reached.

The following observations were recorded:

1. Number of roots.
2. Number of canals per root.
3. Canal configuration (Vertucci’s method).

The canal configuration was classified according to the Vertucci’s method:

1. Type 1 – A single canal present from the pulp chamber to the apex.
2. Type 2 – Two separate canals leave the pulp chamber and join each other to form one canal at the apex.
3. Type3 – One canal leaves the pulp chamber, divides into two canals joining each other to form one canal at the apex.
4. Type 4 – Two separate and distinct canals present from the pulp chamber to the apex.
5. Type5 – One canal leaves the pulp chamber, divides into two separate canals with two apical foramina.

The male and female incidence of second canal was also examined.

## Results

**Figure 1:**
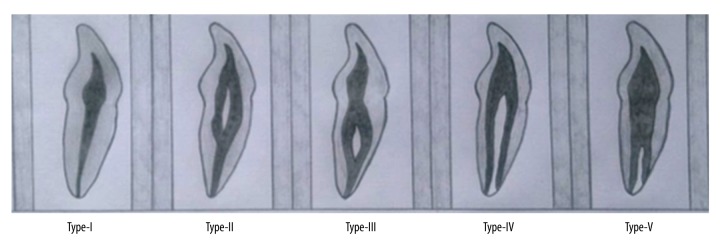
Illustration of five variants of root canal morphology in permanent mandibular incisors according to the Vertucci’s classification.

**Figure 2:**
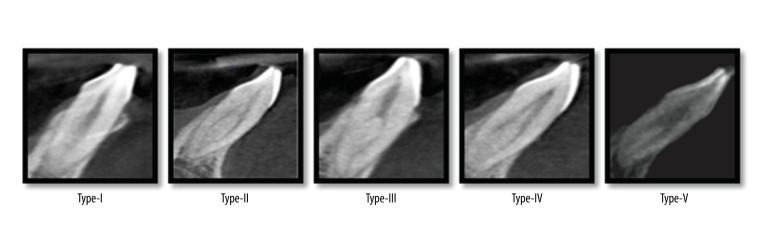
CBCT images showing the five variants in permanent mandibular incisors according to the Vertucci’s classification.

CBCT images of 200 patients (103 males and 97 females) were studied. The patient age ranged from 15 to 60 years. All of the examined teeth were single-rooted. The prevalence of mandibular incisors with a single root canal was 66.5%. Five variants of root canal morphology in permanent mandibular incisors according to the vertucci’s classification are shown in Figure 1. The five variants in permanent mandibular incisors, as visualized by CBCT, are shown in Figure 2. The prevalence of two-canal systems was as follows: right central incisors – 33.5%, left central incisors – 30%, right lateral incisors – 33.5% and left lateral incisors – 36.5% (Tables 1, 2). Type I Vertucci’s configuration (66.5%) was the most prevalent root canal configuration, followed by Type-III (15.25%), Type-II (12.12%), Type-V (3.12%) and Type-IV (2.37%) (Tables 3, 4). According to gender, 15.2% of men and 20.4% of women had a second root canal.

## Discussion

In clinical practice, failure to locate a second canal is the main reason for root canal treatment failure, because it leads to incomplete debridement and obturation of the second root canal. In the present study, all permanent mandibular incisors were single-rooted, which is in consensus with previous studies [13,14]. A literature review reveals that the prevalence of second canal in permanent mandibular incisors varies from 11.5% to 45% [15]. The lowest incidence of 11.5% was reported by Madeira et al. [16], followed by 12.4% in Japan [17], 20% in Greece [18], 25.7% in Vertucci’s study, 29% in the study by Aminsobani, Mukhaimer et al. [19], 36% in the study by Kamtane et al. [20,21] and 45% in the study by Kartal et al. In our study, the incidence of second root canal in mandibular incisors was 33.5%, which is lower than in the study by Kartal et al. in which the prevalence was higher in comparison to the majority of previous studies. These differences are due to different racial origins of the participants, and it also depends on different techniques, sample size, sex and age [20,21].

According to gender, the incidence of second canal was higher in females as compared to males, which is similar to the study conducted by Geduk et al., but in contrast to the study conducted by Liu et al., which suggested a higher incidence of second canal in males as compared to females. The higher incidence of second canal in females gives important information to the endodontist during root canal therapy.

The results of this study also suggested that mandibular lateral incisors had a higher incidence of second canal as compared to mandibular central incisors. Age is also considered as an important factor for the incidence of second root canal, as fewer canals are present due to increasing age associated with the calcification of root canals. It was also reported that increasing age leads to the narrowing and disappearance of root canals [22]. The effect of age on variation of root canal morphology is clear, but age was not analyzed as an influencing factor in the present study.

The strength of this study is the use of CBCT which is associated with a reduced radiation exposure as compared to conventional computed tomography (CT). Moreover, by using CBCT, root canal morphology can be visualized in all the three dimensions. However, a limitation of this study is that it is a retrospective study which was conducted in a limited geographic area, and previous studies suggested that root canal morphology varies between different ethnic groups.

Based on our results, we conclude that CBCT provides three dimensional imaging of dental and maxillofacial structures and is used for making diagnosis in all fields of dentistry. CBCT is associated with lower radiation exposure, faster acquisition time and lower cost when compared to conventional CT. However, CBCT is not a replacement for conventional and panoramic radiography and it is best used for specific diagnosis. The use of CBCT in endodontics for the assessment of complex root canal morphologies, root resorptions and similar conditions is limited [23].

## Conclusions

1. According to this study, mandibular incisors have varied root canal morphology and incidence of second canal was found to be around 33.5%
2. The commonest canal configuration was Vertucci’s Type-1 (Single root canal from the pulp chamber to the apex).
3. The least common canal configuration was Vertucci’s Type-4 (Two separate distinct root canals from the pulp chamber to the apex).
4. Higher incidence of second root canal is found in women than in men.
5. Mandibular lateral incisors have a higher incidence of second canal as compared to mandibular central incisors.
6. CBCT is an excellent imaging modality for the identification of different root canal morphologies.

## Figures and Tables

**Table 1:** Distribution of second canal in mandibular incisors.

No of canal	Left central incisors (31)	Left lateral incisors (32)	Right central incisors (41)	Right lateral incisors (42)
1	140	127	133	133
2	60	73	67	67

**Table 2:** Percentage of second canal in mandibular incisors.

No of canal	Left central incisors (31)	Left lateral incisors (32)	Right central incisors (41)	Right lateral incisors (42)
1	70%	63.5%	66.5%	66.5%
2	30%	36.5%	33.5%	33.5%

**Table 3:** Distribution of the five categories in the root canal anatomy of mandibular incisors

Vertucci’s configuration	Left central incisors (31)	Left lateral incisors (32)	Right central incisors (41)	Right lateral incisors (42)
Type-1	140	127	133	133
Type-2	25	24	19	29
Type-3	28	34	33	27
Type-4	3	8	4	4
Type-5	4	7	11	7

**Table 4:** Percentage of the five categories in the root canal anatomy of mandibular incisors.

Vertucci’s configuration	Left central incisors (31)	Left lateral incisors (32)	Right central incisors (41)	Right lateral incisors (42)
Type-1	70.0%	63.5%	66.5%	66.5%
Type-2	12.5%	12.0%	9.5%	14.5%
Type-3	14.0%	17.0%	16.5%	13.5%
Type-4	1.5%	4.0%	2.0%	2.0%
Type-5	2.0%	3.5%	5.5%	3.5%
